# Oyster farms are the main spawning grounds of the black sea bream *Acanthopagrus schlegelii* in Hiroshima Bay, Japan

**DOI:** 10.7717/peerj.11475

**Published:** 2021-06-01

**Authors:** Kentaro Kawai, Hiroki Fujita, Gustavo Sanchez, Tetsuya Umino

**Affiliations:** Graduate School of Integrated Sciences for Life, Hiroshima University, Higashi Hiroshima, Hiroshima, Japan

**Keywords:** Shellfish aquaculture, Fish spawning, Sparid, Anthropogenic impact, Fish eggs

## Abstract

Understanding the anthropogenic impact of oyster farms is essential for the management and conservation of marine fishes. In Japan, Hiroshima Bay is the region with the most intense oyster farming and thus suitable to study the impact of these farms. Here, we surveyed spherical planktonic eggs of the black sea bream *Acanthopagrus schlegelii*, one of the most abundant fish in the Bay. Our survey was performed at fourteen stations which included places with oyster farms and historical spawning grounds. We found the highest egg densities in four stations, one with historical spawning aggregations and three with major oyster farms. Besides, surveys at the innermost part of Hiroshima Bay, where two major rivers discharge, showed a low density of eggs indicating that black sea bream avoids spawning in low salinity areas. Our study suggests that oyster farms benefit spawners of black sea bream by providing more food sources than historical spawning grounds for efficient spawning. Yet, whether oyster farms represent a full advantage for the species remains unclear, particularly because they are known to host jellyfishes that prey on eggs and limit water flow that can influence the survival of fish eggs.

## Introduction

Hiroshima Bay is an enclosed bay in the western Seto Inland Sea of Japan with an average seabed depth of 25.6 m. The many scattered islands across the Bay make it topographically complex (Kawamura & Shimizu, 1985; Blanco Gonzalez, Umino & Nagasawa, 2008). Water quality in the Bay is strongly influenced by freshwater input from classes A and B of Ohta River and Yahata River located at the most inner part (Hashimoto, Aono & Yamamoto, 2007).

With over 40% of total production in Japan, the aquaculture of the Pacific oyster *Crassostrea gigas* is one of the major profitable industries in Hiroshima Bay (e-Stat, 2020). Oyster-suspended culturing is the most popular method and consists of oyster rafts made of bamboo (8 m wide × 16 m long) and hangs with 600 oyster culturing wires (10 m height) (Kimura & Kaneho, 2003). In the Bay, large-scale oyster farming occurs around Etajima, Noumishima, and northern Ohkurokamishima Islands (Kawaguchi et al., 2011; Yamamoto et al., 2017).

Black sea bream *Acanthopagrus schlegelii* (Sparidae) is an important resource for recreational and commercial fisheries in Japan. Based on genetic analyses, Yamashita et al. (2021) suggested managing this species as a single stock across Japan. Black sea bream is particularly abundant at Hiroshima Bay (Jeong et al., 2007) which is used as a spawning ground, observed based on the higher relative abundances of black sea bream eggs than other fishes (Kawai et al., 2017). Black sea bream is a promiscuous spawner (Jeong et al., 2007) that laid spherical planktonic eggs with diameter ranges from 0.83–0.91 mm during the night-time (Mito, 1963; Koga, Tanaka & Nakazono, 1971). In Japan, the spawning season of black sea bream starts in spring until early summer (April to July) (Yamashita, Katayama & Komiya, 2015; Kawai et al., 2020). Historical spawning grounds of this species are located off western Noumishima Island in Hiroshima Bay (Umino, 2010), where it also faces fishing of bottom-trawling which targets spawning aggregations of this species (Blanco Gonzalez et al., 2009; Umino, 2010). Near the oyster rafts of Hiroshima Bay, the highest density of black sea bream has been reported in May (Tsuyuki & Umino, 2018), which coincides with the peak of its spawning season (Kawai et al., 2020). Here, black sea bream prey on sessile organisms such as mussels, barnacles, and oyster spats of the culturing oyster wires (Saito et al., 2008).

The coexistence of aquaculture industries and a healthy marine ecosystem is a social challenge for the sustainable utilization of marine resources. Oyster farming is one of the most important aquaculture industries worldwide (Forrest et al., 2009). The three-dimensional structure of the farming rafts enhances local biodiversity (Cole et al., 2021) and fisheries production (Peterson, Grabowski & Powers, 2003) by providing habitat and abundance of food for fishes. However, oyster farms also contribute to decreasing of oxygen in benthic environments (Lenihan & Peterson, 1998), limit the water flow (Mutsuda et al., 2011), and form ecological traps for some fishes (Hale & Swearer, 2016; Barrett, Swearer & Dempster, 2019).

The influence of these structures in the spawning ecology of marine fishes remains unstudied. Understanding this influence will contribute to the conservation and sustainability of a productive coastal environment. Hence, here we used a planktonic egg survey of black sea bream eggs to reveal how the largest oyster farming area of Japan influences the spawning ecology of black sea bream.

## Materials & Methods

### Survey of fish eggs

**Figure 1:**
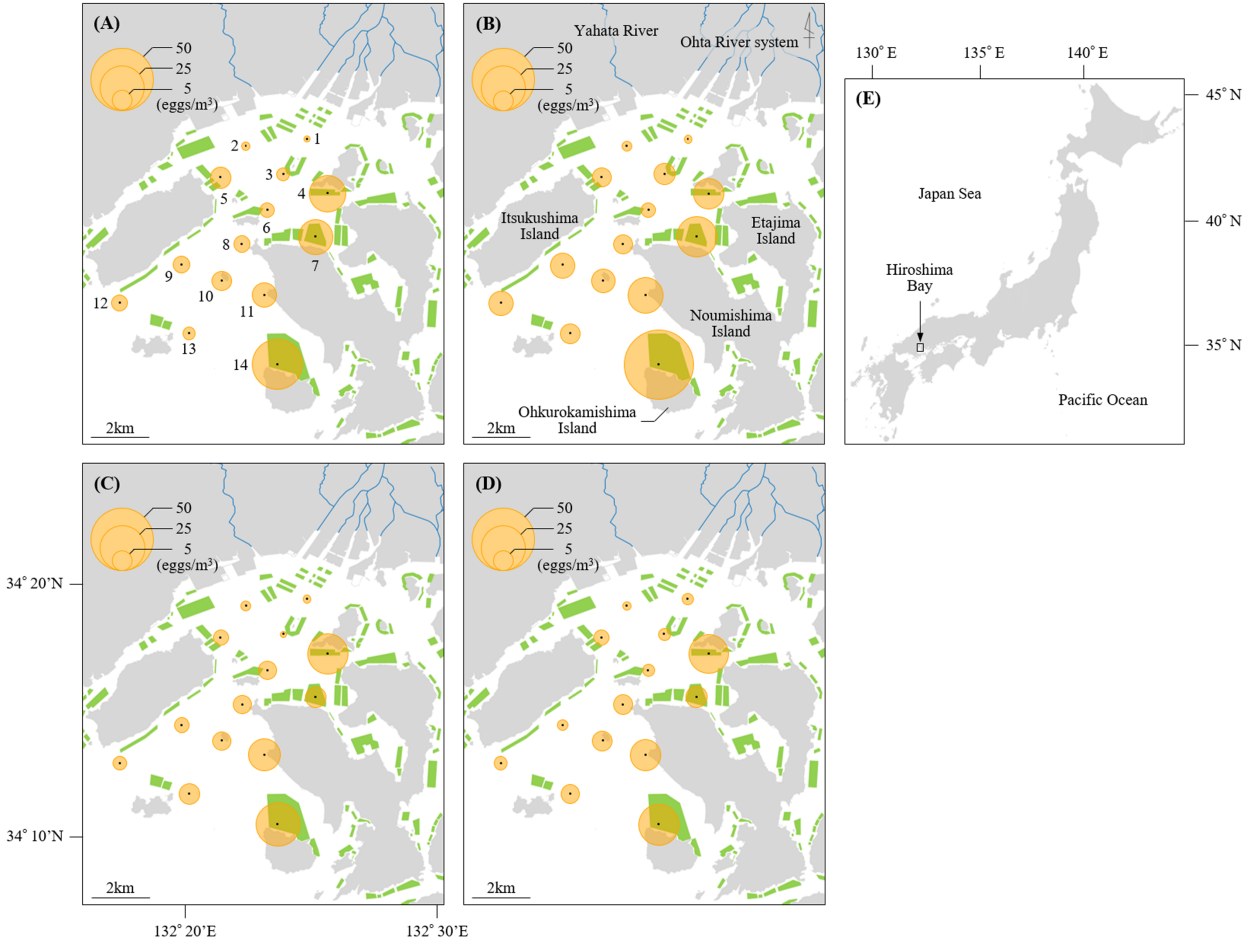
Sampling stations of Black sea bream eggs in Hiroshima Bay. The density of eggs stained positive for black sea bream plotted across the sampling stations at Hiroshima Bay, Japan. The density of eggs at (A) 5 m and (B) 10 m depth in 2016, and at (C) 5 m and (D) 10 m depth in 2017. (E) shows the location of Hiroshima Bay in Japan. Orange circles indicate the densities of black sea bream eggs, and green polygons indicate the size of the oyster farms (available at https://www.pref.hiroshima.lg.jp/soshiki/88/1237480266748.html). Blue lines represent the Ohta River system and Yahata River.

Fish eggs were surveyed at 14 stations (St. 1–14) in Hiroshima Bay from late April to early July of 2016 for seven days, and of 2017 for eight days. These months correspond to the spawning seasons of black sea bream. The sampling locations included large oyster farming areas (St. 4, 7, and 14), around small oyster farming areas, and at two historical spawning grounds (off Noumishima Island) (St. 8 and 11) (Fig. 1). Fish eggs were collected for 5 min at 5 and 10 m depth by using the submersible pump (Tsurumi Mfg. 40PSF2.4S). The pumped seawater volume was calculated as flow per unit time (at 5 m depth was 1.30 × 10^−1^ m^3^/min., and at 10 m was 1.38 × 10^−1^ m^3^/min.). Water quality parameters such as water temperature, salinity, and dissolved oxygen (DO) were simultaneously measured by using an electrical conductivity meter (CM-31P; DKK-TOA Corp., Japan) and a DO meter (DO-31P, DKK-TOA Corp., Japan) at each depth of every station (DO was not measure on late April 2016 because of technical limitation).

We also filtered seawater using a North Pacific standard net (mesh size: 0.335 mm) for screening fish eggs. Spherical fish eggs similar to those of black sea bream (Mito, 1963) were separated and fixed in 80% ethanol for further identification.

We performed surveys at the oyster farms with the permission of the Minou and Mitaka Fisheries Cooperative Association.

### Identification of eggs

Eggs were stained using monoclonal antibodies specific for black sea bream eggs developed in Kawai et al. (2020). The ethanol was removed from eggs by washing them in phosphate-buffered saline (PBS). After that, eggs were immersed in an antiserum of primary antibodies for black sea bream (mouse hybridoma supernatant diluted 100 times) and shook at 37 °C for 30 min. After repetitive washing in PBS, eggs were combined with biotinylated secondary antibodies from a VECTASTAIN Elite ABC Mouse IgG kit (Vector Laboratories Inc., Burlingame, CA, USA) at 37 °C for 30 min.

Eggs were further washed in PBS and incubated with avidin and biotinylated horseradish peroxidase complex at 25 °C for 3 minutes before being stained with diaminobenzidine tetrahydrochloride (Vector Laboratories Inc., Burlingame, CA, USA) at 25 °C for 2–5 min. Finally, eggs of black sea bream that stain reddish-brown were visually counted.

### Data analysis

Densities of black sea bream eggs were calculated by dividing the number of stained eggs by the volume of pumped seawater. Mean egg densities at every station were calculated for each depth and year. For each station, the densities of black sea bream eggs were compared with different depths using the Wilcoxon signed-rank test (*p* < 0.05) implemented in the function wilcox.exact of the R package exactRankTests (Hothorn & Hornik, 2019). These calculations were performed in the Microsoft R Open version 3.5.3 (Microsoft & R Core Team).

## Results

From the total of 3,651 spherical eggs collected, 1,213 out of 2,160 (56.16%) and 735 out of 1,491 (49.30%) eggs stained positive for black sea bream in 2016 and 2017, respectively. The mean densities of black sea bream eggs were 9.19 eggs/m^3^ and 6.35 eggs/m^3^ for 2016 and 2017, respectively.

**Figure 2:**
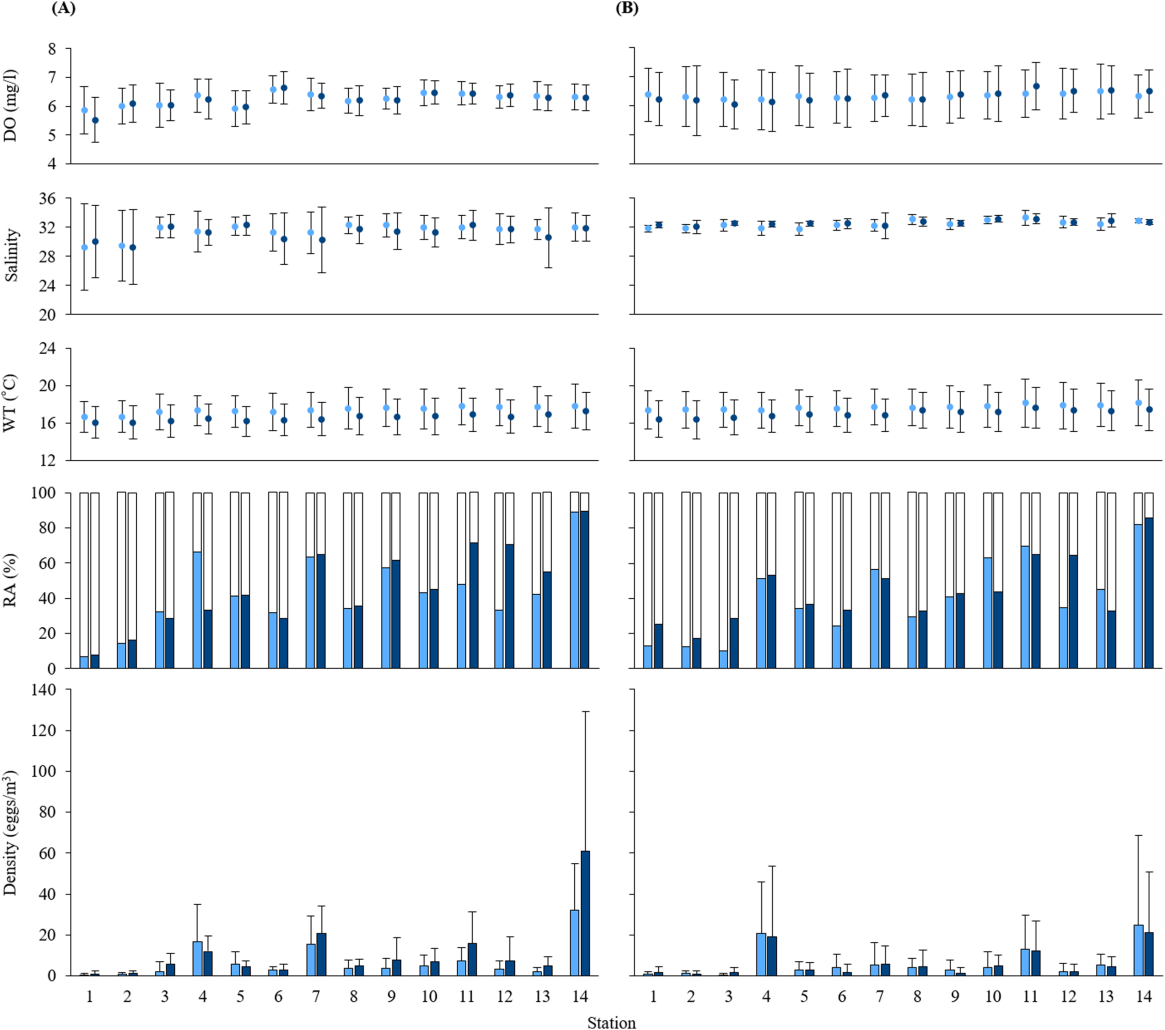
Measurements of water quality. Means of dissolved oxygen (DO), salinity, water temperature (WT), and the relative abundance (RA) and density of black sea bream eggs at each station during (A) 2016 and (B) 2017. Values calculated at 5 and 10 m depth are shown in light blue and dark blue, respectively. Standard deviations are indicated as error bars. White parts in the RA bar graph indicate the non-stained density of eggs.

Mean values of egg densities and relative abundance of black sea bream eggs at each station per year are found in Table S1. Mean egg densities and relative abundance of black sea bream eggs at St. 1 (2016: 0.63 eggs/m^3^, 7.14%; 2017: 1.20 eggs/m^3^, 19.61%) and St. 2 (2016: 1.06 eggs/m^3^, 15.38%; 2017: 1.09 eggs/m^3^, 14.29%) were the lowest compared to the other stations. In contrast, mean egg densities at St. 14 in 2016 (46.61 eggs/m^3^) and 2017 (23.14 eggs/m^3^), St. 4 in 2016 (14.38 eggs/m^3^) and 2017 (20.09 eggs/m^3^), St. 7 in 2016 (18.06 eggs/m^3^) and St. 11 in 2016 (11.71 eggs/m^3^) and 2017 (12.58 eggs/m^3^) were higher than at the other stations. The proportion of black sea bream eggs at St. 14 (2016: 89.45%; 2017: 83.77%) was notably higher than any other stations (less than 74%). Moreover, egg densities did not differ significantly between 5 m and 10 m depth at any station (*p* > 0.05) (Figs. 1 and 2).

The mean values at each depth of water temperature, salinity and DO range from 16.06 to 18.16 °C, from 29.29 to 33.31, and from 5.52 to 6.68 mg/l, respectively. Water qualities of sampling stations were similar at all stations except for the salinity at St. 1 and St. 2. Salinity at St. 1 and St. 2 was around 29, the lowest across all sampling stations in 2016 (Fig. 2). Mean values of all oceanographic conditions per year and per depth are shown in Table S1.

## Discussion

The multiple stages of oocytes observed in the ovaries of mature black sea bream *A. schlegelii* (Law & Sadovy de Mitcheson, 2017) suggest that it acquired the energy to spawn by frequently feeding on external sources. At Hiroshima Bay, large-size adults of black sea bream are abundant aggregates beneath oyster rafts (Sakai, Shimizu & Umino, 2013) to feed on abundant sessile organisms (Saito et al., 2008). These aggregations peaked in May and coincided with the spawning season of this fish (Tsuyuki & Umino, 2018; Kawai et al., 2020). Across the fourteen stations, the relative abundance of black sea bream eggs, as well as the egg density, were higher in major oyster farming areas located at St. 4, 7, and in particular, at St. 14; the latter one of the largest oyster farming area in the Hiroshima Bay (Figs. 1 and 2) (Kawaguchi et al., 2011; Yamamoto et al., 2017). These abundances indicate that black sea bream encounter several food sources at the oyster rafts and has several opportunities for mating and spawn.

We also note that spawning still takes place at historical grounds but in less intensity than at oyster farming areas (Figs. 1 and 2). At the historical spawning grounds, the density of eggs was the highest at St. 11 (around three times more than at St. 8, Fig. 2). Local eggs reduction at historical spawning ground has also been reported for the North Sea cod *Gadus morhua* in the northeast coast of England (Fox et al., 2008), and for the walleye pollock *Gadus chalcogrammus* in the Japan Sea off western Hokkaido, the latter associate with a change in spawning grounds (Miyake et al., 2008). In our study, the difference in egg densities between St. 11 and St. 8 might have been affected similarly as in the case of the walleye pollock.

St. 11 and 8 are also popular for intensive bottom-trawl fishing of black sea bream that targets spawners (Umino, 2010). However, whether intense fisheries activities that target spawners at St. 8 contributes to the decrease of egg abundance requires further comparative assessment using long-term catch per unit effort (CPUE) data.

Oyster farming areas are also near some other sampling stations, at the innermost part of Hiroshima Bay. These farms carried less density and abundance of black sea bream eggs because of their main activity during winter (Mutsuda et al., 2011) rather than the during our sampling period.

No significant difference (*p* > 0.05) was observed in egg densities between 5 and 10 m depth. Since black sea bream is a nocturnal spawner (Koga, Tanaka & Nakazono, 1971; Jeong et al., 2007), these similarities represent the dispersion of eggs until our collection time. Also, the swimming depth of this fish coincides with the wire depths of oyster rafts (Tsuyuki & Umino, 2017) which might also promote the natural homogenization of eggs abundance.

We also found that the density and relative abundance of black sea bream eggs at stations in the innermost part of the Bay (St. 1 and 2) were considerably lower than stations in the outer parts of the Bay (Figs. 1 and 2). These stations receive the freshwater discharge from Ohta and Yahata River, decreasing their salinity. In Kawai et al. (2020), we showed that salinity values lower than 23 produce an abrupt decrease in egg production of black sea bream. Our results suggest that black sea bream avoid spawning in a low salinity environment and does not depend on estuarine environments for spawning. In contrast, timings and places of spawning on other sea bream species such as the riverbream *A. berda* from off South Africa and the pacific seabream *A. pacificus* and the black bream *A. butcheri* from off Australia (Garratt, 1993; Sheaves, Molony & Tobin, 1999; Williams et al., 2013) depend on estuarine environments.

While our study does not assess the development stage of eggs and the effect of tidal currents on their migration, we believe these factors do not significantly influence our main conclusion. Hatchling of black sea bream takes only 40 to 45 h at around 19 °C (Kasahara, Hirano & Ohshima, 1960) and the tidal current in Hiroshima Bay is not fast compared to other open water environments (Kawamura & Shimizu, 1985).

Our study revealed the important role in the coastal ecosystems of oyster farming areas as a new spawning ground for black sea bream. At the same time, these new spawning grounds have reduced the density and abundance of eggs at historical spawning locations. These changes, however, do not necessarily benefit black sea bream. In fact, in recent years, the abundance of black sea bream juveniles, as well as the annual catch of the species, have reduced in Hiroshima Bay (Kawai, Fujita & Umino, 2019; e-Stat, 2020), contrary to the expected increase due to the benefit of oyster farms. Installation of artificial marine structures such as oyster farming areas and the increase of water temperature at the Bay contributes to the proliferation of jellyfishes (Takatsuji, 2003; Lo et al., 2008; Makabe et al., 2014; Boero et al., 2016), one of the main predator of fish eggs (Purcell et al., 1994; Shoji et al., 2010). During our survey, many comb jellies swimming at the oyster farms were also observed. Hence, we believe that while oyster farms might benefit black sea bream dense aggregation of spawners and provides more food than in other nearby locations, they also limit the migration of its eggs to nursery areas (Hinckley et al., 2001; Mutsuda et al., 2011) and might made them easy prey for jellyfishes. These negative effects might increase assuming the continuous climate change and yearly rise of water temperature.

## Conclusions

Our research demonstrates that oyster farming areas influence the spawning ecology of coastal marine fish with planktonic eggs such as the black sea bream, by providing more resources than other areas and becoming main spawning grounds. In our study, we collected black sea bream eggs at 14 stations in Hiroshima Bay, the region with the largest oyster farming area across all the Japanese coast. Our study shows that the densities of black sea bream eggs were higher at oyster farming areas than at the historical spawning ground of this species. However, while oyster farming areas might arrange abundant opportunities for mating and sufficient foods for spawners of black sea bream, they also host jellyfishes and limit water flow which can likely influence the survival rate of fish eggs. In addition, the information collected here is also important for the management of black sea bream in the largest fisheries region of this fish in Japan.

## Supplemental Information

10.7717/peerj.11475/supp-1 Supplemental Information 1 Stational means of densities (Dens.) and relative abundances (RA) of black sea bream eggs, water temperature (WT), salinity and dissolved oxygen (DO) for each sampled depth and year.

## Additional Information and Declarations

### Field Study Permissions

The following information was supplied relating to field study approvals (i.e., approving body and any reference numbers): We performed surveys at the oyster farms with the permission of the Minou and Mitaka Fisheries Cooperative Association, especially Mr. Kei Kubogouchi, Mr. Kenji Nomura, and Mr. Tetsuo Nomura.

### Data Availability

The following information was supplied regarding data availability: Raw data is available at Figshare: Sanchez, Gustavo (2021): Oyster farms in Hiroshima Bay, Japan are used as the main spawning grounds by the black sea bream *Acanthopagrus schlegelii*. Figshare. Dataset. DOI 10.6084/m9.figshare.13729951.v1
